# Developing identities of STEM teachers at emerging STEM schools

**DOI:** 10.1186/s40594-018-0136-1

**Published:** 2018-09-25

**Authors:** Mohamed El Nagdi, Felicia Leammukda, Gillian Roehrig

**Affiliations:** University of Minnesota, 320 Learning and Environmental Sciences, 1954 Buford Ave., St. Paul, MN 55108 USA

**Keywords:** Teacher identity, STEM schools, Case study, STEM teachers, Grounded theory

## Abstract

**Background:**

With the expansion of science, technology, engineering, and mathematics (STEM) schools all over the United States and the world, new roles for teachers are being created, and with these roles, identities are evolving. However, these roles and identities remain an ill-defined area in STEM. The purpose of this paper was to explore the developing STEM teachers’ identities in emerging STEM schools, answering two research questions: (1) How do teachers define their roles as STEM teachers within a STEM school? (2) What do teachers identify as being important characteristics of STEM teachers? A multiple case study design was used to explore the research questions within a bounded context of two emerging STEM schools. Data for this study were drawn from semi-structured interviews conducted with eight teachers from two developing STEM schools within a large urban district in the Midwestern United States. Teams of teachers at each of the schools worked throughout the year to develop and implement their vision for STEM.

**Results:**

Using an inductive data analysis process, three major themes that characterized a STEM teacher identity emerged. These were the unique nature of STEM teachers’ identity; professional characteristics of STEM; and personal characteristics of STEM teachers. Collaboration, flexibility, awareness of students’ needs, and advocates of equity and inclusion were identified as pivotal characteristics of STEM teachers.

**Conclusion:**

This study concluded that STEM teachers’ identity can be viewed as a dynamic, evolving process that results from the interaction of personal and professional traits within new educational experiences exemplified by the STEM endeavor in their schools. An alignment between teachers' personal philosophy and STEM understanding is essential for the success in teaching in STEM schools.

## Background

In order to continue to lead in almost all fields in this globalized world, the USA needs to have high-quality science, technology, engineering, and mathematics (STEM) education (Gonzalez and Kuenzi 2012). However, since the late 1980s, there have been alarming signs that the USA is lagging in performance in international tests, especially in mathematics and science (Forman et al. 2015; National Academy of Sciences 2014). The most recent results show that the USA experienced a three-point drop in average score in mathematics, while remaining relatively flat in reading and science compared to countries of the Organization for Economic Cooperation and Development (OECD) that participated in the 2015 Programme for International Student Assessment (PISA) (OECD 2016). In response, policymakers have called for integrated approaches to K-12 STEM education using authentic learning experiences (National Center on Education and the Economy 2007) to prepare students for the highly competitive twenty-first century with new mindsets and skills (Breiner et al. 2012; Bybee 2010; Morrison 2006; Roberts and Cantu 2012). Consequently, calls for integrating engineering into the K-12 education system, as well as initiating a multidisciplinary framework for in- and out-of-school learning, were the driving force for developing the Next Generation Science Standards (NGSS) (NGSS Lead States 2013).

In spite of the urgency to improve K-12 STEM education at the federal, state, and local levels (Forman et al. 2015) and internationally as well (Ritz and Fan 2015), there has been a sense of vagueness concerning the nature and conceptualization of STEM education not only among teacher educators, but also among other stakeholders, including students, teachers, and policy makers (Breiner et al. 2012; Sanders 2009; Williams 2011). However, it is clear that one of the critical pillars for the success of integrated STEM learning environments, like any other education reform initiative (Wright et al. 1997), is the STEM teacher on whose shoulders STEM integration initiatives come to fruition (Fulton and Britton 2011; Hutchison 2012). In STEM education, “success is brought about by extraordinary teachers who overcome a variety of challenges that stand between vision and reality” (National Research Council 2011, p.19). To be effective, teachers need strong content knowledge and pedagogical expertise (National Research Council 2011; Shulman 1986). In the case of STEM teachers, they require multidisciplinary knowledge across the STEM disciplines and a unique set of pedagogical practices that help design and implement a robust STEM integration curriculum (Kelley and Knowles 2016; Sanders 2009).

It is necessary for STEM teachers to understand the concepts, philosophy, and purposes that an integrated STEM approach entails (Chesky and Wolfmeyer 2015; Breiner et al. 2012) in order to push forward the STEM experiences in their schools. Therefore, as schools move towards adapting and implementing STEM philosophy, it is necessary to decide if one teacher integrates all STEM disciplines into their classes (Hutchison 2012; Sanders 2009) with all the epistemological constraints associated with each discipline (Williams 2011; Sanders 2009), or whether STEM teachers are disciplinary teachers, one for each subject, who collaborate in harmony, developing and implementing STEM-focused curriculum (Herschbach, 2011; Sanders 2009; Williams 2011). Despite this lack of clarity about STEM, STEM-focused schools are opening at an accelerating rate (Forman et al. 2015; Slavit et al. 2016). With the different models of STEM, teachers are left to determine for themselves how to define (a) STEM itself as an approach, (b) the nature of integration, and (c) the role of engineering and technology, and possibly other non-STEM disciplines, in the K-12 system.

As teachers get involved in STEM integration design and practice, they engage in a process of new identity formation of being STEM teachers with the many roles and responsibilities associated with such an identity (Beijaard et al. 2004; Cooper and Olson 1996; Slavit et al. 2016). While there is a strong literature base related to teacher identity across disciplines and grade levels—science, mathematics, elementary, secondary (e.g., Flores and Day 2006; Friesen and Besley 2013; Obenchain et al. 2016)—STEM teachers’ identities have yet to be included within the teacher identity literature. Therefore, research work is critical, especially regarding STEM teachers’ identities and their roles in creating and enacting an instructional and curricular vision of integrated STEM. To fill this gap, this study was initiated and guided by the following research questions: (1) *What do teachers identify as being important characteristics of STEM teachers?* (2) *How do STEM teachers identify their roles as STEM teachers?*

## Literature review

### STEM education

#### Lofty visions and classroom practices

Real-world problems are complex and inherently multidisciplinary. Tackling such problems requires not just the ability to use design thinking or inquiry, but also the ability to choose the best approach or combination of approaches that capitalizes on the strengths of each way of thinking. From this perspective, STEM encompasses the content, skills, and ways of thinking of each of the disciplines, but it also includes an understanding of the interactions between the disciplines and the ways they support and complement each other (Moore et al. 2014). Although educators are aware of the role of STEM education as an economic imperative of education (Chesky and Wolfmeyer 2015; Gonzalez and Kuenzi 2012) as well as a pedagogical need to enhance learning (e.g., Lansiquot 2016; Morrison 2006), neither educators nor researchers consistently agree on definitions for K-12 STEM education or best practices for integrated STEM instruction (e.g., Breiner et al. 2012). However, some common ground has been found as STEM education has evolved into “a meta-discipline, an integrated effort that removes the traditional barriers between STEM subjects, and instead, focuses on innovation and the applied process of designing solutions to complex contextual problems using current tools and technologies” (Kennedy and Odell 2014, p. 246). Unpacking this definition reveals a myriad of tasks and responsibilities for teachers. Integration in curriculum design and implementation, connecting classroom practices with the real world, and focusing on innovation and application are among the tasks that a STEM teacher is expected to fulfill (Morrison 2006). The challenge for teachers lies in aligning such lofty definitions of STEM with their classroom practices, at times creating a dichotomy between theory and practice. Without strong STEM teachers who understand how to embody this vision of integrated STEM, STEM could be reduced to a simplistic version of “design cycles” based on hands-on activities absent of strong science and mathematical content (Williams 2011).

#### Single STEM teacher or team teaching

There are two different visions/models regarding how teachers implement STEM in a school. One point of view treats the “STEM teacher” as someone who can teach science, technology, mathematics, and engineering in spite of all the epistemological variations among these disciplines (Sanders 2009; Williams 2011), eventually creating what might be referred to as a standalone STEM subject or standalone STEM units within a disciplinary course. However, integrative STEM education is not intended as a new standalone subject area in the schools accompanied by new “integrative STEM education” licensure regulations (Sanders 2009). As Sanders (2009) states:

Given the amount of content knowledge necessary to be an effective science, mathematics, or technology educator, it’s very difficult to imagine a new teaching/licensure program that would prepare individual pre- and/or in-service teachers with sufficient science, mathematics, and technology content expertise—and the pedagogical content knowledge—to teach all three bodies of knowledge effectively (p. 21).

Among the barriers that make the alignment between the STEM vision and implementation challenging, especially in the case of the one-for-all STEM teacher, is the epistemological differences between the STEM disciplines.

#### Epistemological constraints

While the NGSS advocate for the integration of engineering into science classrooms, it is important to note that these fields have epistemological characteristics that differ markedly (Herschbach, 2011; Sanders 2009). These characteristics must be fully recognized and accommodated in planning and teaching in order to preserve the intellectual integrity of each field. Otherwise, a very limited understanding undervalues specific intellectual contributions or ignores the collective value of each (Herschbach 2011; Williams 2011). In the following section, a brief delineation of the epistemological differences between science and engineering as a model is explained.

Within a STEM framework, engineering is seen as an umbrella and a context for integration and therefore better learning of science and mathematics (Moore et al. 2014). Engineering can be viewed as either an applied science or a design process. Smith (1988) defined engineering as a design process. He stated:

“[D]esign in a major sense is the essence of engineering; it begins with identification of a need and ends with a product or a system in the hands of a user. It is primarily concerned with synthesis rather than analysis which is central to engineering science. Design, above all else, distinguishes engineering from science” (p. 318).

This perspective of engineering is reflected, to a large extent, in the different STEM integration frameworks used by different researchers and educators in which engineering is viewed as a real-world context for learning mathematics and science, providing a context for developing problem-solving skills, a vehicle to promote the development of communication skills and teamwork, and providing a fun and hands-on setting that will improve students’ attitude towards STEM careers (Moore et al. 2014; Roehrig et al. 2012). Koen (2003) defines engineering as “the use of heuristics to cause the best change in a poorly understood situation within available resources” (p. 28). Heuristics are defined, in this case, as reasonable, plausible, but ultimately fallible approaches; they permit a solution or reduce the time to a solution, but they do not guarantee a solution. Heuristics may include, but is not restricted to, engagement strategies such as cooperative work, holistic perspective versus being lost in minutiae, and better teacher-student interaction (Smith 2006).

While the goal of science is the construction of theories that can provide explanatory accounts of natural phenomena, engineering develops a systematic process for solving problems based on scientific knowledge and models of the material world. Each proposed solution results from a process of balancing competing criteria of desired functions, technological feasibility, cost, safety, esthetics, and compliance with legal requirements. There is usually no single best solution, but rather a range of solutions (National Research Council 2011).

Science begins with a question about a phenomenon and seeks to develop theories that can provide explanatory answers to such questions. In contrast, engineering begins with a problem, need, or desire that suggests an engineering problem that needs to be solved. Engineers use investigation both to gain data essential for specifying design criteria or parameters and to test their designs. Scientific investigations produce data that must be analyzed in order to derive meaning or explanation. Since data usually do not speak for themselves, scientists use a range of tools—including tabulation, graphical interpretation, visualization, and statistical analysis. Engineers also do the same, however, with different purposes. Engineers analyze data collected in the tests of their designs and investigations in order to compare different solutions, determine how well each one meets specific design criteria, and interpret the results (National Research Council 2011).

Given that STEM integration is primarily promoted through reforms in science teaching and learning (Moore 2010; Roehrig et al. 2012), science teachers are at the forefront of most STEM initiatives. Science is, in this sense, taking the lion share of emphasis, causing what might be called “polarity effect” of STEM integration (Roberts and Cantu 2012). In many circumstances, STEM-integrated units are strikingly leaning towards science (Herschbach 2011; Roberts and Cantu 2012; Williams 2011) with little mathematics content, engineering design as a content integrator (context), and technology as a value-laden tool for implementation (Kelley and Knowles 2016).

#### STEM team teaching

One way to address these epistemological considerations is to encourage a team-teaching model as an alternative to a single teacher implementing STEM. In this model, teachers work together and they are able to maintain the epistemological grounding of their discipline. Also, STEM teaching becomes more effective. Student achievement increases when teachers join forces to develop strong professional learning communities in their schools (Fulton and Britton 2011). However, this model requires a great deal of coordination and collaboration among all teachers in both planning and implementation of STEM curriculum (Fulton and Britton 2011; Herschbach 2011). Similarly, Williams (2011) proposed that rather than integration, a more reasonable approach may be to develop interactions between STEM subjects by fostering cross-curricular links in a context where the integrity of each subject remains respected. This approach cannot happen in the absence of a student-centered learning environment.

#### Student-centered learning

While STEM models differ in some regards, there is a common thread related to student-centered learning. In existing models of effective STEM integration, the goal is to provide students with opportunities to construct new knowledge and acquire problem-solving skills through the process of designing artifacts (Bybee 2013; Morrison 2006). This goal is accomplished through a series of open-ended, hands-on activities related to a thematic topic that addresses important concepts related to STEM disciplines (Satchwell and Loepp 2002). Central to this process is involving students in defining and optimizing a solution for a real-world authentic problem from students’ surroundings to help facilitate a more meaningful learning process (Laboy-Rush 2011; Satchwell and Loepp 2002). As a result, students take ownership of their learning and have the chance to make sense of the world rather than learning isolated pieces of information (Morrison 2006). In such an environment, teachers’ roles are critical (Johnson 2012).

#### Teachers’ roles in a STEM setting

Within such a challenging and demanding educational environment, STEM teachers are required to adopt new approaches from the disciplinary approaches they were prepared for. STEM teachers would need to have the content knowledge and professional attributes to organize authentic STEM projects for their students (Laboy-Rush 2011; Morrison 2006). Accordingly, teachers need certain personal and professional traits in addition to the deep knowledge about a broad range of content areas, pedagogical skills across disciplines, and access to appropriate resources (Ruggirello and Balcerzak 2013). In one of the few studies about the characteristics and roles of STEM teachers, Slavit et al. (2016) described the STEM teacher as a “complex mixture of learner, risk-taker, inquirer, curriculum designer, negotiator, collaborator, and teacher” (p.7). While possessing these attributes and engaging in a new and different educational experience, teachers start to develop new identities. As such, teacher identity provides the theoretical framework for this study.

### Teacher identity

Research on teacher identity provides a comprehensive and sophisticated picture of what it means to be a teacher. Exploring the multiple influences that shape teacher identity includes, but is not restricted to, personal experiences, media images, and personal and pedagogical beliefs (Franzak 2002). Hence, developing a teacher identity denotes an ongoing process of construction through professional life that includes social, personal, and professional experiences that happen over an extended time span (Cooper and Olson 1996; Rodgers and Scott 2008). Watson (2006) defines teacher identity as a teacher’s sense of self as a teacher, which encompasses one’s personal, professional, socio-political, and cultural dimensions. Thus, teacher identity is considered a dynamic, continually changing, and active process which develops over time through interaction with different policy, school, and classroom environments and those who work in them (Schutz et al. 2018). Therefore, teacher identity research takes into consideration professional, personal, academic, and social aspects, in addition to teachers’ roles at schools (Beijaard et al. 2004).

#### Social (professional) and personal dimensions of teachers’ identity

Based on reviewing research on teacher identity, there has been a clear dichotomy between looking at teacher identity either as a professional development process through an accumulation of assets, or the growing interest in the inherent personal or individual attributes in each teacher (Beauchamp and Thomas 2009; Kelchtermans 2005; Palmer 1998). The first movement simply focuses on teachers’ acquisition of “assets,” such as knowledge, competencies, and/or beliefs, stressing the importance of desired learning outcomes in terms of “what is learnt” by teachers (Porter et al. 2001). This approach argues that teacher identity formation is a linear process moving from novice to expert. However, this perspective has become debatable given large differences in how teachers develop throughout their career, both between teachers as well as between different expertise areas (Beijaard et al. 2000).

In addition, theories on identity development indicate that development is far from a linear process (Flores and Day 2006). Another limitation of such an “assets” approach is that it perpetuates a discourse about the teacher—the teacher as being the object we look at from above or from the outside. Consequently, such an approach does not allow an understanding of how teachers themselves make sense of their teaching practice (Niessen et al. 2008), thus opening the door for considering inherent, personal dimensions as an integral element in the identity formation process.

Akkerman and Meijer (2011) argue that there are several recurring characterizations of teacher identity. The most commonly seen characterizations are related to three main topics: (1) multiplicity of identity, (2) discontinuity of identity, and (3) the social nature of identity.

The first topic is the multiplicity of identity, which involves “sub-identities.” A teachers’ professional identity consists of sub-identities relating to teachers’ different contexts and relationships.

The second topic is the discontinuity of identity as being “an ongoing process of construction” in which teacher identity is described as fluid and shifting from moment to moment and context to context. Based on their review of literature on teachers’ professional identity, Beijaard et al. (2004) stated that identity is an ongoing process of interpretation and re-interpretation of experiences. Hence, they argued that identity can be seen as an answer to the recurrent question: “Who am I at this moment?” (p. 108). Likewise, Rodgers and Scott (2008) argued that identity is “shifting” and “unstable.”

The third topic is the social nature of identity which relates to various social contexts and relationships in which teachers’ identity develops. Identities are formed in social, communicative contexts and for socially significant reasons (Alsup 2006). Cohen (2010) discussed how teachers negotiate their professional identity in collaborative exchanges, concluding that colleagues constitute key actors in teachers’ formation of professional identity. In other words, Palmer (1998) stated that “identity is a moving intersection of the inner and outer forces that make me who I am” (p.13). Therefore, a more holistic approach towards what it means to be a teacher emerges in which teachers’ professional identity development is understood as involving questions like “who am I as a teacher?” and “who do I want to become?” (Kelchtermans and Hamilton 2004).

**Fig. 1:**
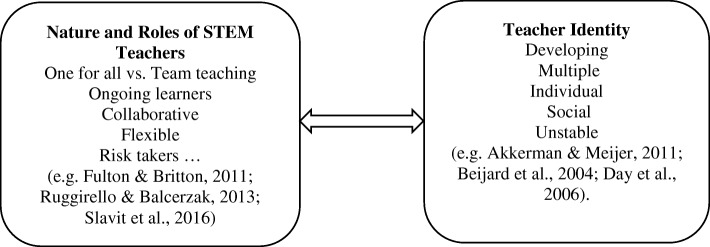
The interaction between assumed roles of STEM teachers and the aspects/attributes of teacher identity

In this study, a teacher’s identity can be looked upon as an outcome of the dialogic relationship between personal and social interaction in a professional setting. Identity is viewed, henceforth, as simultaneously unitary and multiple, continuous and discontinuous, and individual and social. In sum, “identities are a shifting amalgam of personal biography, culture, social influence, and institutional values which may change according to a teacher’s role or circumstance” (Day et al. 2006, p. 613). With such a view in mind, there is a movement in the identity definition and the formation of theoretical discussions from an either/or approach towards thinking in terms of a both/and approach. In other words, a teacher identity is an interaction of both personal and social dimensions as interacting in a reciprocal process of constructing and reconstructing teacher identity (Akkerman and Meijer 2011). Specifically, this study examines how teachers come to understand and identify themselves as STEM teachers, in the professional and personal dimensions. Specific for this study, the nature of STEM teacher identity is conceptualized by interactions between the assumed roles of the STEM teachers in their schools (e.g., Slavit et al. 2016) and the different aspects of teacher identity (e.g., Akkerman and Meijer 2011) (see Fig. 1). Personal dimension attributes represent innate or “self-image” attributes that characterize the STEM teacher’s character or personality, while professional ones refer to “context”-based attributes that help the teacher work in a school setting (Beijaard et al. 2004, p.113). However, at certain points, there is no clear distinction between what is personal and what is professional—there might be some overlap between some of these attributes.

## Methodology

### Research purpose and context

The purpose of this research was to explore STEM teachers’ developing identities in two emerging, urban STEM schools in the Midwestern United States. Subsequently, this exploration can create a contextualized theory as to what constitutes STEM teacher identity (Cooper and Olson 1996) through a grounded theory approach (Charmaz 2014).

The two emerging STEM schools—Falconer and Noddack (pseudonyms)—were part of a larger project with five urban secondary schools in the Midwestern United States that were interested in developing a STEM focus. In each school, a STEM or STEAM (“A” for art) team was created and each school was provided with professional development support, including a STEM education graduate student who served as a classroom coach to help develop STEM curricular units and introduce STEM strategies into the schools (Crotty et al. 2018).

Falconer Middle School is located in a diverse working-class neighborhood. Following a series of school closures in the district, Falconer Middle School recently reopened its doors as a community school with a STEAM focus. The school hired a STEAM coordinator to work towards the goal of being a STEAM school. The student population of Falconer is 72.3% Black, 17.6% Hispanic, 1.8% White, 2.0% Asian, 3.0% American Indian/Alaska Native, 0.5% Native Hawaiian/Pacific Islander, and 2.8% two or more races. The percentage of the student population of Falconer Middle School that qualifies for free/reduced lunch is 87.9% (Minnesota Report Card 2018). At Falconer Middle School, the STEAM team members were appointed by the assistant principal, with one team per grade level—sixth, seventh, and eighth. Each team was expected to create and implement at least one integrated STEAM unit and have students present their work at a community STEAM night.

Noddack is a large middle school located in a historically diverse neighborhood with a highly diverse student population. The population of students who identify as White is 21.4%, while 40.9% are Black (including a large Somali population), 19.7% are Hispanic/Latino, 6.2% are Asian, 9.3% are American Indian/Alaskan Native, and 2.5% are two or more races. The percentage of students who qualify for free/reduced lunch is 76.8% (Minnesota Report card 2018). Membership on the STEM team was voluntary and two teams were formed, based on teacher interest and invitations from each team leader.

The seventh grade STEM experience at Falconer was based on *Project Lead the Way*. It was led by the engineering teacher and science teacher. The sixth grade STEM experience included a unit on American-Indian history and creating dreamcatchers. The eighth grade STEM experience involved students working with local community experts (architects and designers) in redesigning a local park. Students designed and created scaled 3-D models of the park.

At Noddack, one STEM team implemented a unit about the relationships between race, culture, and genetics and how humans are more alike than different. The other STEM team developed and implemented a unit in which students raised chickens in order to learn about sustainability and growth, the scientific method, and computational mathematics for data collection and analysis.

### Research design

The study used a multiple case study design with individual teachers from the two emerging STEM schools representing the case units (Yin 2014). Case study is an appropriate research design for this study due to its intent to explore how teachers in emerging STEM schools identify their roles in a STEM setting. As an “in depth description and analysis of a bounded system” (Merriam 1988, p.40), this case study was delimited by the eight purposefully selected participating teachers in the two schools described above. This study provides a particularistic, descriptive, analytic, and heuristic conceptualization of the topic under investigation: how teachers see their roles as STEM teachers as well as how their identity as a STEM teacher emerges (Merriam 1988; Yin 2014).

### Participants

Eight teachers, four from each of the two middle schools—Falconer and Noddack, described above—were the participants of this study. Participants were purposefully selected for their active participation in the STEM/STEAM team of each school. The participants provided a diverse group of teachers in terms of gender, experience, ethnic background, and age. However, they have a relatively short experience of working in a STEM setting with STEM experience ranging from 2 to 4 years. The teachers were involved in a larger project that introduced STEM curriculum in their schools and participated either voluntarily or by being selected in the STEM/STEAM team in each school. Table 1 shares the demographic information for the participants, the subjects they were assigned to teach, their teaching experience, age, gender, their experience in working in a STEM setting, and school affiliation.

**Table 1:** Teachers’ demographics and experience in both STEM and non-STEM settings

Name^a^	School^a^	Subject and grade level	Ethnicity	Teaching experience (years)	Teaching in a STEM setting (years)	Gender	Age
Betty	Falconer	Science (HS) and engineering (MS)	White	14	4	Female	53
Gary	Falconer	7th grade Life Sciences	Asian	3	2	Male	27
Mary	Falconer	6th/7th/8th grade Art	Latina	28	3	Female	45
Nate	Falconer	8th grade Social Studies	White	13	2	Male	34
Calvin	Noddack	6th grade science	White	4	4	Male	27
Dawn	Noddack	6th/7th/8th grade Art	African-American	20	2	Male	53
Jay	Noddack	Social studies	White	15	4	Male	45
Yen	Noddack	6th/7th/8th grade science	White	13	2	Female	51

^a^Teacher’s names and school names are pseudonyms

### Data collection

Interviews were conducted with the eight teachers at the end of the school year, 1 year into the schools’ development as STEM schools. One of the authors of the study conducted all the interviews with the selected participants at their school sites. The interview questions were the following:

1. What does STEM mean to you? When you hear the word STEM, what comes to mind?
2. Do you identify yourself as a STEM teacher? Why? Why not?
3. How do your ideas about STEM align with your philosophy of teaching?
4. What do you think are important characteristics of a STEM teacher?
5. How is being a STEM teacher different from a (science/math/art/social studies) teacher?
6. How can you see yourself as different from other teachers, if any?

## Data analysis

The interviews were recorded and transcribed. For each of the transcripts, teachers’ responses to the interview questions, developed through the research questions guiding this study, were coded. Content and relational inductive open coding was conducted vertically (for each participant) and horizontally (across the different participants) (Corbin and Strauss 2015; Miles et al. 2013). The coding was done manually and separately at first, and then the authors compared their codes. Alignment of the codes was done, and then an axial coding was used to identify emerging themes. Each respective interview was compared and contrasted with one another.

A cross-case comparison, with each individual teacher being considered a case, was used to synthesize the findings between the separate cases to better understand similarities and differences between them (Corbin and Strauss 2015). These final themes were then compared to previous literature related to teacher identity (Akkerman and Meijer 2011) and the nature of STEM teachers’ roles identified in literature (e.g., Slavit et al. 2016). Through this analysis, three major themes were identified: (1) nature of STEM teachers’ identity, (2) professional characteristics of STEM teachers, and (3) personal characteristics of STEM teachers (Table 2), subsequently, creating a contextualized grounded theory of the characteristics of the developing identity of STEM teachers in emerging STEM schools (Charmaz 2014; Corbin and Strauss 2015; Miles et al. 2013).

**Table 2:** Themes and codes/subthemes identified in the qualitative interviews

Themes	Codes/subthemes
Nature of STEM teacher identity	Developing (emerging) STEM teacher identity Alignment between STEM conceptualization and personal philosophy
Professional characteristics	Believing in equity and inclusion Collaborative with a preference for team teaching Aware of the best practices in STEM Aware of the social issue sand community needs
Personal characteristics	Risk-taker Change agents Open to change Ongoing learning/knowledge seekers

## Results

Three major themes emerged from the data analysis reflecting personal and professional dimensions, as well as a unique nature of STEM teacher identity. The following is a detailed description of the findings.

### Nature of STEM teachers’ identity

The perception of STEM across the eight participating teachers impacted their conceptions of STEM identity in a variety of ways. With the diverse background, age, gender, and subject matter, the teachers developed different conceptualizations of STEM stressing the importance of integration, as well as tackling real-world problems. The alignment between these conceptualizations and their personal teaching philosophies was a critical point for most teachers; they saw that a prerequisite for success in a STEM setting is to have such an alignment. Because STEM teachers came from different subject backgrounds, their ability to identify as a STEM teacher was still unstable. Their identity is in a state of transition, causing multiple identities to form for some teachers being science and STEM, art and STEM, etc. With STEM being a new initiative in their schools, teachers see their identity as STEM teachers as developing and under construction. However, they stressed the importance of having a degree of alignment between their understanding of STEM as a teaching and learning approach and their personal philosophy of teaching.

#### Teachers’ conceptualization of STEM

For all interviewed teachers, STEM was about integration through the addition of engineering and application of knowledge to solve real-world problems to provide an equitable learning experience for all students. As Betty stated, “STEM means to me integrated units. Adding the engineering…It has to be integrated, otherwise you have a STEM class and it’s not STEM education.” Jason added that it is “more real world. [It has] something about the structure [building and design].” Yen stated, “[It is] application of knowledge towards the benefit of society.” Calvin maintains that STEM “support(s) students’ learning” while Dawn stresses that “it is going to be inclusive…to provide equity because some kids...might not learn in the same way.”

Science (as well as social studies and art) teachers who were interviewed argued that STEM provided a more inclusive and holistic learning experience. Yen said that a science teacher is closest to a STEM teacher, but conceded, “instead of being an isolated lab report, [STEM is] solving a problem [that is] very engaging. [STEM teaching] is more relevant [and] engaging, having more focus on equity and cultural relevancy. There is emphasis on the social aspects of science.” Jay believed that STEM teaching “add[s] richness to the experience” of social studies teaching. Meanwhile, Mary thought that being a STEAM teacher added “a particular skill set like engineering, architecture.” However, all of the interviewed teachers still think that their identity as STEM teachers is still under construction.

#### A developing STEM identity (STEM identity under construction)

Seven out of the eight teachers described their STEM identity as still in the making. They were in process of becoming STEM teachers. For instance, Nate, after 13 years of teaching experience and 2 years of working in his present emerging STEM school, he “would identify [himself] as becoming a STEAM teacher.” Mary who has been teaching for 28 years said, “I don’t identify right away as a STEM teacher, but I think I’m working on it.” Yen concurred, “I consider myself as an emerging STEM teacher...not yet a STEM teacher. There is a lot to learn.”

#### Alignment of understanding of STEM and personal teaching philosophy

As experienced teachers, the interviewed teachers come to the STEM setting with their own teaching philosophies. None of them saw any discrepancies between their understanding of STEM and their teaching philosophy. For instance, Yen described a “perfect alignment” between her STEM understanding and teaching philosophy where she thought that linking learning and teaching to everyday life and “extending science experiments to the outside world, not only the lab” was essential for her work as a science teacher. Nate said, “I think being in a STEAM school is right on par with [my philosophy of teaching].” After serving for only 4 years as a science teacher in the same school, Calvin strongly argued that being a STEM teacher “centered me back to what teaching is about for me after feeling burnt out.”

### Professional characteristic of STEM teachers

#### Believing in equity and inclusion

The teachers believed that STEM provides an opportunity for engaging students in the learning process and for bridging the achievement and cultural gaps among different segments of students, including different learning styles, interests, and potential. As stated by Nate, STEM teachers believe that “everybody wants to [and can] learn. I think that it’s our job as educators to bring that out in each child.” Additionally, Yen believed in the role of STEM in “engagement and bridging gaps among students.”

Teachers unanimously considered student learning as the focus of all the teaching/learning processes in a STEM context. Not only should teaching/learning be the focus, but mentoring as well. Yen emphasized the importance of “mentoring students in schools to get exposure to different STEM fields...while collaborating with other teachers.” Jay believes that “the much more important things my students should learn is problem-solving, being okay with failing.” Jay went as far as to be “happy to spend money ‘to help kids’ projects’.” Referring to content coverage versus student learning, Yen said that “[Teachers shouldn’t be] caught up in a checklist of what needs to be accomplished, but rather giving our children the space and time to inquire.”

#### Awareness of social issues and connections between school and community

Participants believe that STEM leads to community service and solving community problems. Four teachers emphasized that teachers should be aware that they are helping the community surrounding their schools, and understand the community from all the different aspects—culturally, socially, economically, and even politically. Yen believed that “[STEM] emphasizes the social aspects of science. [STEM is] application of knowledge towards the benefit of society.” Betty stated, “Teachers need to understand what [STEM] means for that community and then participate in it.” Mary, who lives in the same district where most of the students come from, gave a very powerful understanding of her role. She stated, “I am [from this community] and I live here...I want to give something to these kids that they normally wouldn’t have. [I’m]…passionate about exposing them to as much as possible -- all the possibilities I have at their fingertips… It’s my community; I feel responsible. I love these kids and I can’t say that everybody feels the same way.” Betty, on the other hand, believed in “trying to make [curriculum] more relevant for our kids or stretching it out to teach them the things they don’t know.”

#### Collaborative and a preference for team-teaching

Collaborative planning with teachers of other subjects in order to get exposure to different STEM fields was among the most recurrent themes of the teachers interviewed. “One of the main features of a STEM teacher is the ability to work with others,” said Dawn. Mary emphasized that it is important to “plan with other teachers…we work as a collaborative team…integrate science into art for problem-solving purposes. So much of science is bigger than just an experiment.” Calvin went as far as to state that “the best way…to see a STEM teacher is to be with a group of teachers. I don’t think there’s a stand-alone STEM teacher…[what is needed is a group of teachers with] a very mixed and diverse academic background.”

#### Aware of STEM best practices (problem- and project-based learning)

Project-based learning and problem-based learning were considered as the backbone of STEM education by the participants (e.g., Morrison 2006; Moore 2010). The interviewed teachers concur with this fact. According to Nate, STEM teachers work on “using best practices and [identify] what has worked before in other STEAM schools, like problem- and project- based learning which are seen as integral parts of STEM education.” Betty elaborated, “I guess the part that it aligns is that it’s more problem-based. We used to call it inquiry-based [in science], but I think it’s getting more specific…if we add STEM, it’s going to have rigor versus if it’s just inquiry-based.” Yen agreed with Betty saying that, “problem-based learning is an inherent part [of] science; however, when students redesign what I do through project-based learning, they learn more as they understand what they experience more.”

### Personal characteristics of STEM teachers

#### Risk takers, change agents/open to change

One of the most critical characteristics of STEM teachers, according to the participants, is being open to change and being change agents at the same time. Betty thought that the main characteristics of a STEM teacher is the ability to be “flexible and open for change,” while Jay argued that seeing failing as an opportunity for learning and “problem-solving” were critical components of a STEM teacher’s character. Gary thought being “risk takers” while working in a “sustainable place to do this” is essential for the success of STEM teachers.

#### Ongoing learners, knowledge seekers

Teachers talked about different attributes that put the teacher in the place of the learner; they are ongoing learners, thirsty for professional development and research. Once a STEM teacher presented himself/herself as a guide and a model to his/her students, many of the characteristics needed in students had to be exemplified by the teacher. “It is a learning process,” Yen said*.* Likewise, Jay said, “Put the teacher in the role of the learners,” while Gary conceded that he is still “a student.” Mary said that she has more to learn. She explained, “those kinds of things you might have to develop and learn a little bit more, but I still think that as an art[ist] and learning about the arts, you’re kind of again…bringing in history or bringing in science.” According to Dawn, [STEM teachers] should have the “ability to research to find the other connections between the different forms of different technology” that would help them in their work as STEM teachers. Jay summed it up by saying “a STEM teacher should have the same characteristics a STEM student is required to have.”

## Discussion

Our data reveals that STEM teacher identity is not a simple construct and there are several factors at play as teachers work towards integrating STEM in their classrooms. STEM teachers described their identity development as a journey of ongoing learning, collaboration, awareness of the community needs, and alignment of their own teaching philosophy with the requirements of a STEM approach. These conceptualizations concur with the reviewed literature on teacher identity (e.g., Akkerman and Meijer 2011; Franzak 2002; Schutz et al. 2018) where the nature of teacher identity is viewed as a dialogical concept where personal and professional experiences interact to create an emerging STEM teacher identity in the context of this study.

Both at the personal and professional levels, teachers interviewed in this study provided a clear picture of how they see themselves as STEM teachers in a developing STEM setting. Viewing themselves as emerging or developing STEM teachers fits within the conceptual underpinnings of teachers’ identity (e.g., Akkerman and Meijer 2011; Mockler 2011; Watson 2006) per the ongoing process of identity building through different contexts and experiences. Teachers present what can be described as a “fluid identity,” where the new STEM experiences they are involved in through professional learning and creating integrated STEM experiences for their students add a new dimension to their existing teacher identity. While these new STEM experiences create a higher level of commitment and motivation for the teachers to go through the new journey of identity formation, they, in turn, explain how experienced teachers serving more than 20 years of teaching see themselves as developing STEM teachers. This aspect concurs with the argument of Rodgers and Scott (2008) that states that identity is “shifting” and “unstable.” Day et al. (2006) concomitantly note that “identities are a shifting amalgam of personal biography, culture, social influence, and institutional values which may change according to one’s role or circumstance” (p. 613). This is exemplified by what the participants had to say in the interviews; Mary, for instance, mentioned she was “working on it [developing her identity as a STEM teacher].”

Viewing the educational experience through wider lenses is another aspect of the emerging identity of the teachers in these emerging schools. STEM is seen as providing a wider perspective than individual disciplines such as science, social studies, and art. The teachers see themselves as providers of a more holistic learning experience to their students. For instance, though Yen referred to herself as a science teacher, she instantly conceded that STEM is more comprehensive and “more relevant and engaging” if compared to the science silo where the teacher is “isolated in the science lab.”

As identity formation is viewed as an outcome of the interaction of what is personal/individual and what is professional/social (Akkerman and Meijer 2011; Day et al. 2006), participating teachers stressed the need for an alignment between what they personally believe as a teacher and the philosophy of integrated STEM education. This alignment between what is personal and what is professional/social provides another layer of teacher identity that could provide answers to questions such as “Who am I as a [STEM] teacher now?” and “What do I want to become?” (Kelchtermans and Hamilton 2004). There was a unanimous emphasis among all teachers interviewed on the necessity of such an alignment. The interviewed teachers denote on different occasions in their responses that there is an alignment between their personal teaching philosophy and their conceptualization of STEM. For instance, Yen described this alignment as “perfect”, while Nate stated that a STEM approach is “right on par with [his philosophy of teaching].” Inversely, when a teacher’s philosophy is unaligned with the STEM requirements of ongoing learning and inclusive teaching, there would be a problem. This misalignment would not only adversely affect the professional community, but would also cause problems for both students’ learning and eventually to himself/herself as an educator.

The job of a STEM teacher is challenging given the wide range of requirements at different levels, including curriculum design, implementation, being aware of the best practices in the field, and having an eye on social issues (Morrison 2006; Hutchison 2012; Ruggirello and Balcerzak 2013; Slavit et al. 2016). This scope necessitates that STEM teachers, from the perspective of our participants, possess certain characteristics, including being an ongoing learner, being open to change, looking at failure as an opportunity for learning, and believing in the need to provide equitable and inclusive learning opportunities to all students. Given the growing need for STEM teachers for the advancement of STEM programs all over the country and the world (National Research Council 2011), understanding the critical characteristics of STEM teachers is an important part of advancing to that goal.

Teachers emphasized the need for collaborative work in a sustained manner in order to help build equitable and inclusive learning experiences for students. They see collaboration, mainly in the team-teaching model, as vital for the success of a STEM teacher. The need for integration and interaction of different disciplines to solve problems is a basic feature of STEM education (e.g., Bybee 2013; Herschbach 2011; Moore 2010). If a teacher is not collaborative or is reluctant to engage in ongoing professional learning to develop his/her teaching skills, he/she will be a big hurdle for the STEM team that requires higher levels of collaboration and ongoing learning (Wang et al. 2011). Not only does teacher collaboration support student learning, teachers who work in strong learning communities are more satisfied with their careers and are more likely to remain in teaching long enough to become accomplished educators (Fulton and Britton 2011). Therefore, developing a collaborative professional atmosphere in a STEM setting is one of the prerequisites for a successful STEM integration (e.g., Fairweather 2008). This integration is usually done in a framework of flexibility and openness for change. Betty, for instance, thought that the main characteristics of a STEM teacher was the ability to be “flexible and open for change”, while Calvin and Dawn believed that a huge feature of a STEM teacher was “being capable of communicating with colleagues” (Hutchison 2012; Ruggirello and Balcerzak 2013; Slavit et al. 2016).

## Conclusions

In this study, we explored the developing identity of STEM teachers in emerging STEM schools. Teacher identity, as explored in this study, reflects a dynamic nature; it is developing and changing in nature. Teachers see themselves as learners, still on the road to becoming full STEM teachers, equipped with different dispositions on the personal and professional level. Developing a STEM teacher identity can be viewed as marathon not a sprint.

In the words of one of the STEM teachers, “a STEM teacher should have the same characteristics a STEM student is required to have.” This statement is true at all levels—personal and professional. STEM teachers need to be flexible, open to change, collaborative, problem solvers, and aware of the recent trends in teaching and learning. In addition, STEM teachers have a teaching philosophy aligned with their understanding of STEM education. This alignment reduces the internal conflict between what a teacher believes and what he/she is required to do in addition to decreasing the external conflict with other stakeholders while grappling with the process of implementing a STEM focus.

## Implications

This research contributes to the literature of STEM teacher identity and better understanding of the changing roles of teachers in a STEM school. Understanding the necessary characteristics of STEM teachers informs decision making for teacher preparation, recruitment, and selection to work in STEM schools. This understanding, in essence, would help promote teacher performance leading to higher levels of student achievement while reducing internal and external conflict for teachers working in a STEM school (Fulton and Britton 2011; Slavit et al. 2016; Wang et al. 2011). We must keep in mind the high demand for well-prepared STEM teachers (National Research Council 2011) and the need for teachers who can align their personal teaching philosophies to STEM requirements, who behave with a learner mindset, who are problem solvers, and who are collaborators.

People in leadership roles in STEM schools can benefit from the results of this study at different levels. First, they need to believe genuinely in the ability of STEM to make changes in students’ learning, and not an opportunity for a catchy title or funding. Second, in terms of teacher selection, clear criteria for selection where the different characteristics of STEM teachers discussed in this paper, such as alignment of personal teaching philosophy and requirements of STEM, flexibility, and openness for collaboration, are rigorously utilized. These criteria can be established by using structured interviews and/or class observations in the early stages of recruitment. Third, while implementing STEM curriculum in the school, school leaders should show the needed support in terms of an understanding of what it takes to implement STEM curriculum and provide the necessary guidance and timely professional development programs within a collaborative professional learning community. In brief, a STEM school principal should have the same or similar characteristics to be found in STEM teachers and/or students. A STEM school principal “can very well be the provocateur, the [one who] leverage[s], the agent of change, and the one who opens the door, inviting and supporting new thinking and new practice into his or her school” (Myers and Berkowicz 2015, p. 31).

## Future research

This study was based on interviews with teachers working in emerging STEM schools regarding their assumptions and conceptions of the roles and identity formation as STEM teachers. More research is needed in order to understand if and how these reported characteristics of a STEM teacher corresponds to actions in their classrooms and the impact of these conceptions on students’ interest and learning in STEM disciplines. Also, more work is needed in order to explore the discrepancy between existing teacher preparation programs and STEM requirements. Six out of the eight teachers indicated that teacher preparation was not preparing qualified STEM teachers who can implement the required teaching and learning strategies, and above all, have the personal and professional characteristics to be STEM teachers.

## Abbreviations

NGSS: Next Generation Science Standards
OECD: Organization for Economic Cooperation and Development
PISA: Programme for International Student Assessment
STEAM: Science, technology, engineering, art, and mathematics
STEM: Science, technology, engineering, and mathematics
